# Prevalence for the Disclosure of HIV Status to Sexual Partners and Its Determinants among Adults under cART in Amhara Region, Northwest Ethiopia

**DOI:** 10.1155/2022/9941380

**Published:** 2022-07-07

**Authors:** Awoke Seyoum Tegegne

**Affiliations:** Bahir Dar University, Department of Statistics, Bahir Dar, Ethiopia

## Abstract

**Background:**

Globally, the transmission of HIV from one individual to another causes 1.8 million new infections each year, 36.7 million people living with HIV, and one million people died from HIV-related illnesses. The objective of this study was to determine the prevalence of the disclosure of HIV status to sexual partners and its determinants among adults under cART in the Amhara Region, northwest Ethiopia.

**Methods:**

A retrospective study design was conducted on 792 randomly selected samples. The study was conducted in the Amhara Region, from 2015 to 2020. A binary logistic regression modeling was used for data analysis. The data were collected using a stratified random sampling technique where the residential areas were considered strata. Data were collected by trained health practitioners in the ART section in Felege Hiwot Teaching and Specialized Hospital. The hospital is a referral in which many patients from different districts and zonal hospitals in the region are referred to this hospital.

**Results:**

The rate of disclosure of HIV status to sexual partners in this study was 21%, which is very low compared to the average rate of disclosure in developing countries. Among the predictors, age of patients (AOR = 1.02, 95% CI:(1.001,1.120); *p*-value = 0.004); number of baseline CD4 cell count (AOR = 0.980; 95% CI: (0.764, 0.991); *p*-value<0.01); number of hospital visits (AOR = 1.01; 95% CI: (1.001, 1.034); *p*-value < 0.01); marital status (living with partner) (AOR = 1.01; 95% CI: (1.003, 1.112); *p*-value = 0.006); female HIV-positive adults (AOR = 1.01; 95% CI: (1.001, 1.021); *p*-value = 0.007); rural residence (AOR = 0.98; 95% CI: (0.96, 0.99); *p*-value = 0.004); non-educated adult patients (AOR = 0.950, 95% CI: (0.92. 0.98); *p*-value = 0.003); cART non-adherent adult patients (AOR = 0.940, 95% CI: (0.61. 0.97); *p*-value < 0.001); non-opportunistic infectious diseases (AOR = 1.062, 95% CI: (1.049. 1.191); *p*-value = 0.002); and non-existence of social violence (AOR = 1.012, 95% CI: (1.008, 1.); *p*-value < 0.01) significantly affected the variable of interest. Of these, the number of CD4 cell count, male HIV-positive adults, rural residence, and existence of social violence negatively affected the variable of interest.

**Conclusions:**

Some groups of HIV patients did not disclose their level of HIV status to their sexual partners. Health-related education is recommended for patients who did not disclose their HIV status to sexual partners. This helps to reduce the transmission of HIV from infected individuals to noninfected ones and from mother-to-child HIV transmission.

## 1. Introduction

Currently, HIV/AIDS continues to be a serious global public health problem. It is the cause of 36.7 million people living with HIV and 1.8 million new infections each year. The problem is also the cause of one million people dying from HIV-related illnesses [1]. Among these, about 19.4 million people are testified to live with HIV in Eastern and Southern Africa [2].

In Ethiopia, the problem seems to be stable given that it is different in different regions in the country. According to the Ethiopian Public Health Institute (EPHI) report, the Amhara Region, one of the eleven regions in the country, accounts for the highest number of people living with HIV. In the region, the overall incidence rate of new HIV infection is 6.9 per 1000 tested population [3].

Several factors are responsible for reducing the infection, which can be grouped as economic, social, and demographic factors. Individual behavior is the most significant factor in one's casual of acquiring the infection [4].

One way of reducing the spread of the disease may be encouraging people living with the virus to disclose their disease status to their sexual partners [5]. This may be important to reduce the transmission of HIV by making awareness and decreasing risky behavior [6].

Disclosing one's HIV status to sexual partners means speaking fairly about sexual alignment, possible drug-taking practice, and results of tests. These are often unthinkable issues that are hard to talk about openly and honestly in most communities [7].

Some people living with HIV may generally hide their HIV status from people in their lives, including their sex partners. In addition, individuals living with the virus who do not disclose their HIV status may have had bad experiences related to previous disclosures [8]. Some of the experiences may be loss of social support, not being employed by different organizations, violent reactions, and other forms of discrimination [9]. People who do not disclose their HIV status may lack a sense of worth for being able to effectively disclose their disease status, especially to sex partners [10].

Self-disclosure of the HIV disease status generally has important effects on an individual's health, lower stress, and leads to better psychological relief [11]. In the case of HIV/AIDS, individuals who disclose their HIV status are in better health conditions in terms of reproductive choices as well as psychosocial support [12].

Disclosure of the HIV status facilitates other behaviors that may improve the management of HIV. Previous studies indicate that individuals who disclosed their HIV diagnosis results have better adherence to ART treatments [13]. Female adults who disclose their HIV status to sexual partners are more likely to participate in the prevention of mother-to-child HIV transmission programs [14]. Studies previously conducted indicate that disclosure may increase opportunities to receive social support, which may help individuals cope and recover from physical illness, and decrease depressive symptoms due to HIV-related indications [13]. Disclosure of HIV-positive status to all societies living around them is crucial for HIV avoidance and care execution policies. Hence, it is important to discover the prevalence of disclosure of HIV status to sexual partners and its factors determining individuals not disclose their HIV-positive status in order to reduce the transmission of the disease to uninfected people.

Among studies conducted previously in the developed world, rates of the disclosure of HIV disease to sexual partners ranged from 42% up to 100%, depending on the large part on the type of partner to whom the person disclosed. The previous studies also indicate that the rate of disclosure of the disease in developing countries is lower than the rates reported in developed countries. The rates of disclosure in developing countries vary from 16.7% to 86% with average disclosure of 49% [15].

To the best of the author's knowledge, there is limited region-wide research on the prevalence of disclosure of HIV status to sexual partners and its predictors among HIV-positive adults under cART.

The issue of disclosure of HIV status increases opportunities for implementation of HIV risk reduction, improving access to treatment, and motivating partners for Voluntary Counseling and Testing (VCT) activities [15]. Thus, disclosure of HIV status is an issue to be addressed for HIV prevention and treatment [13]. The objective of this study was to determine the prevalence of disclosure of HIV status to sexual partners and its associated factors among adults living with HIV/AIDS (PLWAs) in the Amhara Region, Ethiopia. The result obtained in the current investigation is important for regional policymakers to make evidence-based HIV prevention and interventions.

## 2. Methods and Materials

### 2.1. Study Area and Population

The study was conducted in the Amhara Region (northwest Ethiopia). The region is one of the nine well-known regions in the country with a large population which is the second next to the Oromia region. The region has 12 zones, three-city administrations, and 180 woredas (139 rural and 41 urban). According to the Ethiopian Central Statistics Agency, the region has a projected population of 21.5 million people, about 80% of whom are rural farmers. The region had only 80 public hospitals, 847 health centers, and 3,342 health posts. Amhara's healthcare system is unable to modernize and provide quality health services due to many challenges particularly; the transmission rate of the disease from one individual to another is still a series of problems. This is why the region was selected as a study area. The study population in the current investigation was all HIV-positive adults under treatment.

### 2.2. Study Design

A retrospective cohort study design was conducted on 792 randomly selected adult HIV-infected patients under cART in the Amhara Region, Northwest Ethiopia. In the hospital, about 6 thousand people with HIV were receiving treatment and of these, about 2 thousand of them were under cART. The data were taken in ART sections of Felege Hiwot Teaching and Specialized Hospital and its catchment areas. The hospital is a specialized, teaching, and referral with a regional laboratory, where all HIV patients throughout the region are referred to this hospital and all treatment results are sent to this hospital for a regional laboratory experiment. Finally, the regional laboratory results are organized and sent to the Federal Ministry of Health.

### 2.3. Source of Data

Secondary data collected from participants' charts by the health staff for treatment purposes were used in the current investigation. A binary logistic regression model was used for investigating the variable of interest.

### 2.4. Participants

The source populations for the current investigation were all HIV-positive adults under cART and following their treatment at zonal and district hospitals and treatment results were sent to Felege Hiwot Teaching and Specialized Hospital, Amhara Region, Ethiopia. The study population was adults who fulfilled the inclusion criteria.

#### 2.4.1. Sample Size and Sampling Procedures

Random samples of 792 HIV-positive adults were selected considering their ART identification number. Cochran's formula was conducted in determining the sample size [16,17]. The sample size is taken at each district, and the zonal hospital was selected using a stratified random sampling technique considering their residential area as strata. Finally, a random sample of patients at each zonal and district hospital was selected using a systematic random sampling technique, considering the patient ID in the hospital.

Cochran's formula is used for calculating the sample size when the population is large. Cochran (1977) developed a formula to calculate a representative sample for proportions as

(1) $$\begin{array}{c} n_{o}=\frac{z^{2}pq}{e^{2}}, \end{array}$$

where *n*_*o*_ is the sample size, *z* is the selected critical value of desired confidence level, *p* is the estimated proportion of an attribute that is present in the population, *q* = *p* − 1, and *e* is the desired level of precision. For a population whose degree of variability is unknown, *p*=0.5, *q* = 0.5, and *e* = 0.05. Taking 99% confidence level with ±5% precision, the calculation for the required sample size would be *p*=0.5 and hence *q* = 1 − 0.5 = 0.5; *e* = 0.05; and *z* = 2.58. So,

(2) $$\begin{array}{c} n=\frac{2.58^{2}\left(0.5\right)\left(0.5\right)}{{\left(0.05\right)}^{2}}=665.6=666. \end{array}$$

In the sample size determination formula, two conditions were considered:

- the more the sample size is, the more accuracy that one can get and
- missingness and non-response rate

Because of the above conditions, an investigator added about 20% more participants in the study and it becomes 792.

#### 2.4.2. Inclusion Criteria and the Study Period: HIV-Infected

Patients under cART with at least two visits to the treatment site whose follow-ups were from January 2015 up to December 2020 were included in this investigation. Hence, the study period was from 2015 up to 2020.

#### 2.4.3. Variables under Current Investigation

The dependent variable for this study was disclosure of the HIV status to sexual partners among HIV-positive adults under cART. It has two levels namely disclosed and not disclosed. The disease is said to be disclosed if a sexual partner had full information about the status of the disease, otherwise, it is not disclosed. Since the patients considered under this investigation are under cART (combined antiretroviral therapy), a patient is said to be adherent if the three are conducted correctly.

The predictor variables were sex, age, marital status, level of education, social support, social violence, residential area, the existence of mental depression, religion, functional status, opportunistic infectious disease, WHO stages of HIV, adherence levels, and baseline CD4 cell count. The categories of each predictor are indicated in Table 1.

#### 2.4.4. Self-Reported Predictor Variables

In addition to the above predictors, variables like dietary instruction, the time when pills were taken, the existence of mental depression, the existence of social violence by people living together, the existence of social support, and the existence of medication allergic at the initial time were reported by participants and recorded carefully in each patient's chart.

In the current investigation, defaulters were patients who did not come back to the ART clinic until the end of the study period (31 December 2020). A defaulter could be existed as a result of death, transfer to another hospital, and loss-to follow-ups [18].

At the initial time of the treatment, patients were directed to visit the hospital monthly for the first 6 months and quarterly for the remaining study period. Hence, there were 23 follow-ups for those patients with full visits of observation in the study period. The reason for monthly follow-ups at the initial time was to follow up on whether there existed medication side effects like mental depression, skin scratch, and any other medication allergic on individuals at the initial time.

### 2.5. Data Analysis

Data were edited, cleaned, coded, and entered into a computer and analyzed using SAS software. Descriptive statistics were conducted to assess basic participants' characteristics. Bivariate analysis was conducted to determine the presence of statistically significant correlations between explanatory variables and the outcome variable. Model selection was assessed using the stepwise selection technique. Odds ratios (OR) and their 95% CI were also used to look into the significant effect between the dependent and independent variables.

In this investigation, a person was categorized as food adherent if he/she always followed dietary instructions directed by the health staff, otherwise, he/she was categorized as non-adherent (self-reported food adherence). Similarly, a patient was categorized as time adherent if he/she always followed time scheduling instructions given by the health practitioners otherwise categorized as non-adherent. Patients' self-report on whether drug medication had been skipped or not were used to assess adherence to medication. Based on this, a person was said to be non-adherent to medication, if he/she took <95% of the prescribed pills. If a patient's adherence is ≥95% of the prescribed medication, he/she is categorized as adherent to medication.

Hence, for comparison purposes, a combined indicator of adherence (cART) was made using the three adherence measures taking into account all questions pertaining to adherence. So, in the current investigation, non-adherence was defined as a PLWHA missing any one of the three criteria mentioned above (medication, time, and dietary).

### 2.6. Data Collection Tools and Quality of Data

The data collection tools/format were developed by the investigator in consultation with the health staff at the ART section of Felege Hiwot Teaching and Specialized Hospital, and the quality of data was controlled by the health staff at the ART section. To assure the quality of the data, the questionnaire was pretested on PLWHA (5% of the sample size i.e., 40 individuals) and amendments were incorporated to the questionnaire to obtain full information on the variables included in the investigation.

Statistical Analysis System (SAS**)** version 9.4software was used to analyze the data. A binary logistic regression model was employed for the longitudinal outcome variable (disclosure of the HIV status of their sexual partner). A statistical decision was made at a 5% level of significance.

### 2.7. Models Used in the Current Investigation

In this investigation, an analysis of binary data in terms of the binomial distributions with logit transformation was conducted. The result is a binomial response conducted with a logistic regression model with a logit link function.

First, the binary random variable Y with probabilities P(*Y* = 1) = *π* and P(*Y* = 0) = 1−*πwas considered*. Then recalling the random variable, Y has the binomial (*n*, *π*) as:

(3) $$\begin{array}{c} P\left(Y=y\right)=\;\left(\begin{array}{c} n\\ y \end{array}\right)\pi^{y}{\left(1-\pi \right)}^{n-y},\;y=0,1,2,\ldots n, \end{array}$$

where *π*_*i*_ depends on a vector of observed covariates **x**_i_ and let *π*_i_ be a linear function of the covariates as *πi = β*_*0*_*+β*_*1*_*X*_*1*_*+…+βkX*_*k*_.

The probability *π*_i_ has to be between zero and one, but the linear predictor can take any real value. The solution for such a problem is to model the transformation as a linear function of the covariates, and it is done as follows:

First, the probability *π*_*i*_ to the odds was defined as odds _i_ =   *π*_*i*_/(1 − *π*_*i*_)

*Second*, the calculation for the logit or log-odds was expressed as

Log it (*π*_*i*_) = log(*π*_*i*_/(1 − *π*_*i*_)) =  *β*_0_+*β*_1_*x*_1_ +------+*β*_*k*_*x*_*k*_.

The above formula has the effect of removing the floor restriction. This is known as the logistic regression model that follows a linear model. In this model, the effect of a unit change in *X*_j_ is to increase/decrease the log-odds by an amount *β*_j_ keeping the other predictors constant. Equivalently, the model may be written in terms of the odds of a positive response as:

(4) $$\begin{array}{c} \pi_{i}/\left(1-\pi_{i}\right) \end{array}$$

= exp (*β*_0_ +*β*_1_*x*_1_ +------+*β*_*k*_*x*_*k*_).

Here the effect of a unit change in *X*_j_ is to increase/decrease the odds of a positive response by the factor exp (*β*_j_) called the odds ratio.

Finally, the probability of a positive response was considered as

(5) $$\begin{array}{c} \pi_{i}=\frac{\exp\left(X_{i}^{\prime }\beta \right)}{1+\exp\left(X_{i}^{\prime }\beta \right)}. \end{array}$$

### 2.8. The Goodness of Fit

The goodness of fit for the current investigation was conducted using the Akaike information criterion (AIC) and Bayesian information criterion (BIC), considering the model with the smallest AIC and BIC as the best of all others.

### 2.9. Operational Definitions

Sexual partners: People who engage in sexual activity together. The sexual partners may be in a committed relationship, either on an exclusive basis or not, or engaged in sexual activity on a casual basis.

## 3. Results

The baseline characteristics of participants are indicated in Table 1.

As shown in Table 1, out of the sample of 792 patients, 40.9% were rural residents, 50.6% were females, 56.3% were living with their partners, 21% disclosed their disease to family members, and 49.2% were owners of cell phones. Only 25.5% of the patients were adherent and the rest were non-adherent. Finally, among the respondents, less than one-third of the patients disclosed their disease status to sexual partners (21%).

Among the participants who disclosed their HIV status, 17.3% disclosed the disease status on the day of receiving the test result, 18.5% disclosed their status within a week, 9.7% of them disclosed their disease status within 2 weeks, and the remaining of them disclosed their disease status within a month.

Reasons for non-disclosure of the disease status were recorded by the health staff and some of the reasons were: 35% as fear of separation/divorce, 37.7% of them said that their partner might be afraid of the transmission of HIV from them, 25.5% of the other said fear of accusation of disloyalty, 7.1% of the participants not disclose because of fear of being labeled as a bad person, 5% of them said that no enough time to discuss because their partner works in other place, and 6.1% declared that because of fear of physical abuse. About 59.8% of respondents expected a partner's support before disclosure.

As shown in Table 1, out of the sample of 792 patients, 40.9% were rural residents, 50.6% were females, 56.3% were living with their partners, 21% disclosed their disease to family members, and 49.2% were owners of cell phones. Only 25.5% of the patients were adherent and the rest were non-adherent. Finally, among the respondents, more than 50% of them (79%) did not disclose the disease to sexual partners.

Among the participants who disclosed their HIV status, 17.3% disclosed the disease status on the day of receiving the test result, 18.5% disclosed their status within a week, 9.7% of them disclosed their disease status within 2 weeks, and the remaining them disclosed their disease status within a month.

Reasons for non-disclosure of the disease status were recorded by the health staff and some of the reasons were; 35% as fear of separation/divorce, 37.7% of them said that their partner might be afraid of the transmission of HIV from me, 25.5% of the other said fear of accusation of disloyalty, 7.1% of the participants not disclose because of fear of being labeled as a bad person, 5% of them said that no enough time to discuss because my partner works in other place and 6.1% declared that because of fear of physical abuse. About 59.8% of respondents expected a partner's support before disclosure.

Parameter estimation which helps to identify statistically significant predictors for the variable of interest is indicated in Table 2. Table 2 indicates that predictors like age of patients, baseline CD4 cell count, the number of followed-up visits, marital status, sex, residential area, opportunistic infectious diseases, level of education, and level of adherence to cART had a significant effect on the variable of interest.

As the age of patients increased by 1 year, the expected odds of being disclosed the status of HIV to a sexual partner was increased by 2% assuming that the other things remain constant (AOR = 1.02, 95% CI: (1.001,1.120); *p*-value = 0.004). However, as the number of baseline CD4 cell count increased by one cell/mm^3^, the expected odds of being disclosed the status of HIV disease to sexual partners was decreased by 2%, keeping the other covariates constant (AOR = 0.980; 95% CI: (0.764, 0.991); *p*-value < 0.01).

As the number of hospital visits increased by one unit, the expected odds of being disclosed the HIV status to a sexual partner was increased by 1%, keeping the other conditions constant (AOR = 1.01; 95% CI: (1.001, 1.034); *p*-value < 0.01).

Table 2 also indicates that marital status had a significant effect on the variable of interest. Hence, comparing patients living with their partners with those living without partners, the expected odds of being disclosed the HIV status of a sexual partner for patients living with their partner was increased by 1% keeping the other covariates constant (AOR = 1.01; 95% CI: (1.003, 1.112); *p*-value = 0.006).

The expected odds of being disclosed the HIV status to sexual partners for female adults was increased by 1% as compared to males, keeping the other things constant (AOR = 1.01; 95% CI: (1.001, 1.021); *p*-value = 0.007).

The predictor variable, residential area, significantly affected the disclosure of the HIV status of sexual partners. Hence, the expected odds of being disclosed the HIV status for sexual partners by the rural HIV-infected adults was decreased by 2% keeping the others constant (AOR = 0.98; 95% CI: (0.96, 0.99); *p*-value = 0.004.

The expected odds of being disclosed the HIV status for sexual partners by non-educated adult patients was decreased by 5% as compared to educated adults, keeping the other things constant (AOR = 0.950, 95% CI: (0.92. 0.98); *p*-value = 0.003).

Similarly, the expected odds of being disclosed the HIV status for sexual partners by cART non-adherent adult patients was decreased by 6% as compared to cART adherent adults, keeping the other things constant (AOR = 0.940, 95% CI: (0.61. 0.97); *p*-value< 0.001).

The existence of social violence had a statistically significant effect on HIV-positive adults not disclosed their status with HIV disease to sexual partners. Hence, the expected odds of being disclosed the HIV status for sexual partners by HIV-infected individuals, where there is no social violence, was increased by 1.2% as compared to those HIV-infected adults living in the societies, where there is social violence, keeping the other things constant (AOR = 1.012, 95% CI: (1.008, 1.234); *p*-value < 0.01).

The expected odds of being disclosed the HIV status for sexual partners by non-opportunistic disease adult patients was increased by 6.2% as compared to opportunistic adults, keeping the other things constant (AOR = 1.062, 95% CI: (1.049. 1.191); *p*-value = 0.002).

WHO stages had also a statistically significant effect on the disclosure of the level of the HIV status of sexual partners. Hence, the expected odds of being disclosed the HIV status by adult patients whose WHO stage 1 was decreased by 11.3% as compared to WHO stage 4 keeping the other variables constant. Similarly, the expected odds of being disclosed the HIV status by adult patients whose WHO stage 2 was decreased by 12.2% as compared to WHO stage 4 keeping the other variables constant, and the expected odds of being disclosed the HIV status by adult patients whose WHO stage 3 was decreased by 9.5% as compared to WHO stage 4 keeping the other variables constant.

## 4. Discussion

This study tried to identify the intensity/prevalence of disclosure of HIV status and its predictors among HIV-positive adults under cART. The prevalence of this study indicates that among the total participants, only 21% of them disclosed their HIV status to their sexual partners. This indicates that the prevalence was very low as compared to the average rate of prevalence conducted in other developing countries (49%). The potential reason for this might be cultural, social, and economic factors. Potential predictors have been identified for different levels of disclosure of the disease status as discussed below. This needs further study.

Age significantly affects the level of disclosure of the HIV-positive status for people living with HIV. As age increases, the disclosure levels of the disease status to sexual partners also increase. It is known that sexual intercourse decreases as age of an individual increase and this may encourage disclosing the disease to sexual partners. Hence, being older, the HIV-infected individuals are likely to have a steady sexual partner, and this contributes to an increase in the rate of disclosure [6,19]. Another previously conducted research indicates that the younger age group may not go for HIV testing, and such people may not disclose their status unknowingly [20].

HIV-positive people with a high number of CD4 cell counts feel comfortable and healthy as compared to those with a low number of CD4 cell counts; such people consider themselves HIV negative and they need not accept the diagnosis result given by the health staff. Hence, they are not volunteering to disclose their HIV status. The result in this regard is consistent with another previously conducted investigation [5].

As visiting time of the heath institution increase, HIV-positive adults are encouraged to disclose their disease status because of their awareness and health-related education they got during every visiting time at health institutions. This result is similar to another previous research [21]. When HIV-positive adults visit the health institution as prescribed by the health staff, such people might be exposed to other individuals during visiting, and communication with such people encourages them to disclose the disease [22].

Marital status also significantly affects the degree of disclosure of HIV status. HIV-positive adults living with their partners increase their willingness to disclose the status of their disease as compared to adults living without their partners. The potential reason for this might be the fact that adults living with their partners fill more concerned about the care of partners. Disclosure of the disease for adults living with partners might help each other as a reminder to take the pills on time and also important to remind the date when the partner should visit the health institution. The result in this regard is similar to previously conducted research [7] and contradicted another research [23]. Hence, this result needs further investigation. Disclosure of HIV status to sexual partners empowers couples to make knowledgeable reproductive health varieties that may ultimately lower the number of unplanned pregnancies among HIV-positive couples and even reduce the risk of HIV transmission from mother to child [24].

Female HIV-positive adults are more likely to disclose their disease status to their partners as compared to males. The possible reason for this might be the fact that males need multiple partners as compared to females. Such needs discourage males to disclose their disease status and they need to hide the disease. Another reason may be the fact that females are willing to disclose their HIV status, due to the responsibility of their concern for their partners' health or to avoid their guilt [8]. This result is supported by previous research [25] and contradicted with another investigation [26]. The reason for the contradicted result is that females hide their disease because of their fear of stigma and discrimination [26]. This result also needs further investigation.

Urban HIV-positive adults are more likely to disclose their disease status as compared to rural HIV-positive adults. Urban patients might have a better understanding of disclosing the disease to get social support from the government and communities around them [27]. The culture in rural areas is more strict as compared to urban, and the HIV-positive adults who disclosed their disease status might be discriminated against by the society because of the reason that societies in rural areas lack information on how and when the disease is transmitted from one individual to another [27].

Social violence has a significant effect on the HIV people not disclosed their disease status to their sexual partners. The potential reason for this might be that HIV-infected adults fear the trend that those individuals disclosed the disease violated by people living together [12].

Education plays a significant role in the variation of disclosure level of HIV status. Educated people are more likely to disclose the disease to sexual partners [28]. The potential reason for this might be the fact that such people have more information about the use of disclosing the disease to society, especially to their sexual partners [29]. Knowledge on how to prevent HIV transmission is important to disclose the HIV status and this encourages disclosing the disease to sexual partners [29].

## 5. Conclusion

Only a few of the participants (21%) under investigation disclosed their HIV status to their sexual partners which is a creditable strategy that will target those not likely to disclose and will have to be evolved. Overall, the level of disclosure of HIV-positive results in this study is below the rate of disclosure status at developing (49%) countries. This indicates that more health-related educational work is needed to rise up the disclosure level of the HIV disease. Different patients disclosed the status of the disease at different times and only 17.3% disclosed the disease status on the day of receiving the test result.

Important predictor variables had been identified for the difference in levels of the disclosure of HIV disease status. Among the predictors, age of patients, follow-up visits, living with partners, female patients, non-existence of social violence, non-opportunistic disease, and being educated patients were positively associated with the increase of disclosure of the HIV disease status, whereas the existence of social violence, being non-adherent to cART, non-educated patients, male patients, living without partner, and baseline CD4 cell count were negatively associated with disclosure of HIV disease status.

As a recommendation, health-related education for HIV-positive adults to disclose their HIV status is a crucial issue. Knowledge of HIV transmission is also important to reduce the violence and discrimination against those HIV-positive adults who disclosed their disease status. Special support for that HIV-infected individual who disclosed the disease may encourage the others to disclose their disease status without fear and anxiety.

This research was not without limitations. The data were taken in one treatment site, and the other treatment sites may provide additional information about the prevalence and predictors associated with why HIV-infected individuals do not disclose their HIV status to sexual partners, friends, and relatives and generally to the society.

## Figures and Tables

**Table 1:** *Baseline sociodemographic and clinical variables of 792 participants in the study area*.

Variable		Average (Q1, Q3)	No (%)
Weight (kg)		58.1 (45–70)	—
Baseline CD4 cells/mm^3^		148.7 (113–180)	—
Age (years)		64.3 (48–78)	—
Follow-up times		23 visits	—
First month/initial CD4 cell count change/mm^3^		16.6 (12–26)	—
Sex	Male		392 (49.4)
	Female		400 (50.6)
Educational status	No education		163 (20.6)
	Primary		209 (26.4)
	Secondary		274 (34.6)
	Tertiary		146 (18.4)
Residential area	Urban		468 (59.1)
	Rural		324 (40.9)
Existence of social violence	Yes		345 (56.4)
	No		447 (43.6)
Existence of mental depression	Yes		478 (60.4)
	No		314 (39.6)
Availability of social support	Yes		350 (44.2)
	No		442 (55.8)
Marital status	Living with partner		446 (56.3)
	Living without partner		346 (43.7)
WHO HIV stage	Stage I		101 (12.8)
	Stage II		259 (32.7)
	Stage III		199 (25.1)
	Stage IV		233 (29.4)
Disclosure of the disease	Yes		166 (21.0)
	No		226 (79.0)
Adherence to cART	Adherent		202 (25.5)
	Non-adherent		590 (74.5)

**Table 2:** Parameter estimates for disclosure of HIV status for sexual partners.

Parameter	Estimates	St. error	Adjusted odds ratio (AOR)	Wald 95% CI		*p*-value
Intercept	3.01	0.03	20.287	11.53	58.62	<0.001^*∗*^
Age	0.02	0.01	1.020	1.001	1.120	0.004^*∗*^
Baseline CD4 cell count	−0.02	0.01	0.980	0.764	0.991	<0.001^*∗*^
Follow-up times	0.01	0.01	1.010	1.002	1.034	<0.001^*∗*^
Marital status (Ref. = Without partner)						
With partners	0.01	0.021	1.010	1.003	1.112	0.006^*∗*^
Sex (Ref. = Male)						
Female	0.01	0.012	1.010	1.001	1.021	0.007^*∗*^
Residential area (Ref. = Urban)						
Rural	−0.02	0.023	0.980	0.96	0.99	0.004^*∗*^
Level of education (Ref. = educated)						
Non-educated	−0.05	0.452	0.950	0.92	0.98	0.003^*∗*^
Adherence (Ref. = adherent)						
Non-adherent	−0.06	0.471	0.940	0.71	0.97	<0.001^*∗*^
Existence of social violence (Refer = yes)						
No	0.012	0.354	1.012	1.008	1.234	<0.001^*∗*^
Opportunistic infectious disease (Ref. = yes)						
No	0.06	0.521	1.062	1.049	1.191	0.002^*∗*^
WHO stages (Ref. = stage IV)						
Stage I	−0.12	0.347	0.887	0.645	0.921	0.013^*∗*^
Stage II	−0.13	0.065	0.878	1.12	1.05	0.021^*∗*^
Stage III	−0.10	0.048	0.905	0.09	1.10	0.010^*∗*^

^*∗*^stands for significant variables at 95% CI.

## Data Availability

The data used for the current investigation are available from the corresponding author

## Abbreviations

PLWHA: People living with human immunodeficiency virus
AOR: Adjusted odds ratio
CI: Confidence interval
HIV: Human immunodeficiency virus
cART: Combined antiretroviral therapy
WHO: World Health Organizations
AIC: Akaike information criterion
BIC: Bayesian information criterion
SAS: Statistical analysis system.
